# Integrated Omic Analyses Reveal Module Networks Regulating Growth and Bioactive Component Synthesis of *Sophora tonkinensis* via Calcium Modulation

**DOI:** 10.3390/plants15010133

**Published:** 2026-01-02

**Authors:** Zhu Qiao, Zhan-Tao Fan, Ling-Yun Chen, Lin-Xuan Li, Fan Wei, Shuang-Shuang Qin, Jing Wang, Ben Qin, Ying Liang

**Affiliations:** 1 Guangxi Key Laboratory of Medicinal Resources Protection and Genetic Improvement, Guangxi Engineering Research Center of TCM Resource Intelligent Creation, National Center for TCM Inheritance and Innovation, Guangxi Botanical Garden of Medicinal Plants, Nanning 530023, China; qiaozhu@gxyyzwy.com (Z.Q.); lilinxuan1125@163.com (L.-X.L.); weifan@gxyyzwy.com (F.W.); qinss@gxyyzwy.com (S.-S.Q.); wangjing@gxyyzwy.com (J.W.); qinben@outlook.com (B.Q.); 2 Jiangsu Key Laboratory of TCM Evaluation and Translational Research, China Pharmaceutical University, Nanjing 211198, China; fanzhantao_zwy@163.com; 3 School of Traditional Chinese Pharmacy, China Pharmaceutical University, Nanjing 211198, China; lychen@cpu.edu.cn

**Keywords:** *Sophora tonkinensis*, transcriptome, proteome, metabolome, medicinal plant, calcium

## Abstract

*Sophora tonkinensis* is a key medicinal plant endemic to the calcium-rich karst regions along the China–Vietnam border. This study investigated how calcium regulates the growth and biosynthesis of bioactive compounds in *S. tonkinensis* tissue culture seedlings by exposing them to a gradient of calcium concentrations. Our findings demonstrate that a moderate calcium level (T2, 2.99 mmol·L^−1^) promoted root development, increasing root dry weight, and significantly elevated the content of matrine and oxymatrine. In contrast, a high calcium level (T4, 5.98 mmol·L^−1^) inhibited root growth, reducing root length, but triggered a distinct metabolic shift, markedly increasing the accumulation of trifolirhizin and maackiain. Integrated transcriptomic, proteomic, and metabolomic analyses revealed that calcium exerts systemic regulation through multiple functional pathways. We identified four key signaling pathways—phytohormone, plant immunity, MAPK, and phospholipid signaling—as central hubs coordinating genetic information processing, vesicular transport, and metabolic reprogramming. These results provide valuable insights into the calcium-mediated regulatory networks and offer valuable targets for optimizing cultivation practices to enhance the yield of bioactive compounds in *S. tonkinensis*

## 1. Introduction

*Sophora tonkinensis*, a prominent medicinal plant, is primarily found in the karst rocky mountains along the tropical northern border of China and Vietnam, an area with soil naturally rich in calcium. The plant’s growth environment suggests that calcium plays a pivotal role in determining its medicinal properties [1]. With a long history of use in traditional Chinese medicine, *S. tonkinensis* contains pharmacologically active compounds, specifically quinolizidine alkaloids such as matrine and oxymatrine, as well as isoflavonoids like maackiain and trifolirhizin, all of which exhibit significant pharmacological activities [2]. However, the mechanisms through which calcium regulates the synthesis of these medicinal components remain unclear.

Calcium plays a multifaceted role in plant growth and development, acting both as an essential nutrient and a key signaling molecule [3]. It is well-established that calcium acts as a key signaling agent, triggering the production of calcium-binding proteins in plants [4], which rapidly and precisely modulate intracellular calcium levels, thereby amplifying its signaling function [3]. As a central signaling component, calcium regulates diverse cellular processes [5] and physiological functions such as pollen tube growth [6,7], plant responses to pathogens and symbiotic microorganisms [6,8,9], tissue injury responses [10,11], and mineral element absorption [12,13]. These processes are closely linked to broader physiological outcomes, including metabolism, growth, and development. Consequently, variations in calcium concentrations are likely to influence the medicinal compound content of *S. tonkinensis*. Given the broad functional scope of calcium, it is hypothesized that it modulates the synthesis of pharmacological components through a complex network of signaling and metabolic pathways, rather than through a single linear route.

Elucidating calcium’s role in secondary metabolite synthesis using conventional genetic approaches remains challenging, particularly for non-model medicinal plants with limited genomic resources and long life cycles. To address these limitations, the integration of multi-omics technologies and bioinformatic analysis has been increasingly recommended as an effective strategy [14,15,16]. This approach helps overcome key research bottlenecks such as long experimental cycles, scarce genetic information, and the absence of target metabolites in common model plants.

The recently completed whole-genome sequencing of *S. tonkinensis* provides a solid foundation for in-depth molecular analysis. Based on the diverse roles of calcium and its potential multi-pathway influence on medicinal components, this study employs an integrated transcriptomic, proteomic, and metabolomic approach to delineate functional modules and their interactions under calcium exposure. This methodology is well-suited for unraveling complex systemic mechanisms in biological systems. Several recent studies have successfully applied multi-omics strategies in medicinal plant research, as demonstrated in *Dendrobium officinale* [17], *Glycyrrhiza uralensis* [18], *Perilla frutescens* [19], and *Catharanthus roseus* [20].

To investigate the calcium-mediated regulatory networks governing growth and bioactive compound accumulation in S. tonkinensis, this study analyzed phenotypic and phytochemical responses under a series of calcium concentrations. A comprehensive multi-omics analysis was further employed to uncover the underlying molecular mechanisms through which calcium influences these processes.

## 2. Results

### 2.1. The Impact of Calcium on the Growth and Development of Tissue-Cultured Seedlings

**Figure 1:**
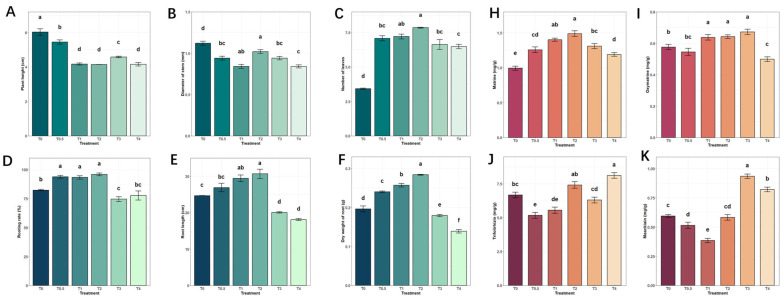
Effects of varying calcium concentrations on the growth and development of tissue-cultured plantlets of *S. tonkinensis* and their bioactive compound content (values followed by the same letter are not significantly different at *p* < 0.05): (**A**–**C**) growth status of the aerial parts; (**D**–**F**) growth status of the root parts; (**H**–**K**) bioactive compound content in the plantlets.

The growth responses of S. tonkinensis tissue-cultured seedlings to exogenous calcium were evaluated across a concentration gradient (T0: 0 mmol·L^−1^, T2: 2.99 mmol·L^−1^, T4: 5.98 mmol·L^−1^; see Table 1). As shown in Figure 1A–C, calcium supply suppressed aerial growth, resulting in reduced plant height and stem diameter relative to the control (T0), but increased leaf number. Root development, in contrast, exhibited a concentration-dependent response (Figure 1D–F). The T2 treatment promoted root growth, as indicated by higher rooting rate, longer roots, and increased root dry weight, whereas the T4 treatment strongly inhibited these parameters.

This study focused on four key active components of *S. tonkinensis*: matrine and oxymatrine (quinolizidine alkaloids) and trifolirhizin and maackiain (isoflavonoid derivatives). The results revealed that matrine and oxymatrine levels were significantly elevated under T1, T2, and T3 calcium treatments compared to the control. Under T4 calcium treatment, however, matrine levels decreased, though they remained higher than in the control group, while oxymatrine levels were significantly lower than in the control group. Trifolirhizin content significantly increased under T4 treatment, while maackiain levels were markedly higher in both T3 and T4 treatments. Conversely, trifolirhizin and maackiain levels were lower than the control under T0.5 and T1 calcium treatments (Figure 1H–K).

### 2.2. Initial Examination of Transcriptome Data

Omics data were collected for treatments T0, T2, and T4, as these had the most pronounced effects on growth and pharmacological profiles in *S. tonkinensis* tissue-cultured seedlings. The T2 treatment enhanced root development (Figure 1D–F) and increased matrine and oxymatrine levels (Figure 1H,I). In contrast, T4 treatment reduced root length (Figure 1E) and dry weight (Figure 1F), while also decreasing oxymatrine levels (Figure 1I), but it significantly increased trifolirhizin and maackiain levels (Figure 1J,K). The T0, T2, and T4 treatments were selected for subsequent multi-omics profiling as they represented the control baseline, the most growth-promotive, and the most metabolic shift-inducing conditions, respectively, thereby providing the strongest contrasts to elucidate key regulatory networks.

**Figure 2:**
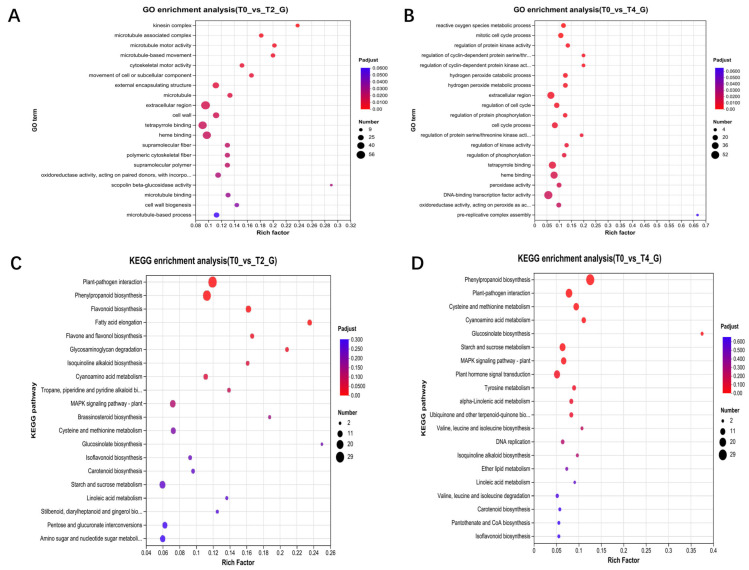
Enrichment analysis of differentially expressed genes from transcriptomic data: (**A**,**B**) GO functional enrichment analysis of differentially expressed genes in T2 and T4 compared to T0; (**C**,**D**) KEGG functional enrichment analysis of differentially expressed genes in T2 and T4 compared to T0.

Transcriptome analysis identified 3282 differentially expressed genes (DEGs) between T2 and the control, with 2402 genes upregulated and 808 downregulated in T2 (Supplementary Figure S1A). Similarly, 2588 DEGs were identified between T4 and the control, with 1901 genes upregulated and 687 downregulated in T4 (Supplementary Figure S1B). A comparison of T4 and T2 revealed 1491 DEGs, with 740 genes upregulated and 751 downregulated in T4 (Supplementary Figure S1C). Gene Ontology (GO) annotation indicated that DEGs under T2 treatment were primarily enriched in functional modules related to vesicle transport, metabolism, and cell wall regulation (Figure 2A), while DEGs under T4 treatment were predominantly associated with metabolism, cell cycle regulation, and signal transduction (Figure 2B). KEGG pathway analysis demonstrated that DEGs under T2 treatment were significantly enriched in various metabolic and signal transduction pathways, particularly those related to plant immune responses (Figure 2C). In contrast, DEGs under T4 treatment were enriched in similar metabolic pathways and additionally in DNA repair processes (Figure 2D).

To further investigate the KEGG pathways involved in differential gene expression, Gene Set Enrichment Analysis (GSEA) was conducted. The GSEA results for differential genes between T2 and T0 (Supplementary Figure S2A), T4 and T0 (Supplementary Figure S2B), and T4 and T2 (Supplementary Figure S2C) are shown, with the specific functions of each pathway listed in Table 2.

**Figure 3:**
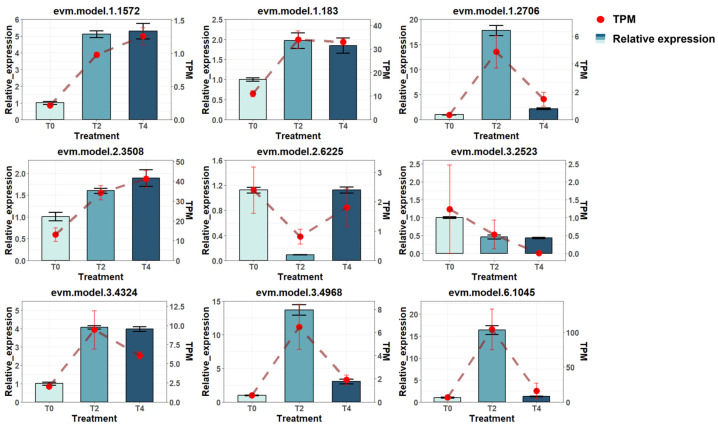
Validation of gene expression consistency between real-time PCR results and transcriptomic data for selected genes. TPM: transcripts per million, a unit representing gene expression levels derived from transcriptomic data; Relative expression: gene expression levels quantified by RT-PCR experiments.

The transcriptome-based gene expression data were validated through real-time polymerase chain reaction (real-time PCR) of nine randomly selected genes. The expression levels determined by PCR were compared with the TPM estimates at T0, T2, and T4 (Figure 3). The results confirmed that the gene expression trends obtained from the transcriptome data were consistent with those verified by the experimental method.

Proteomic analysis revealed significant protein content differences between T2 and T0. Among 310 proteins, 182 were upregulated and 128 downregulated in T2 compared to the control (Supplementary Figure S1D). Similarly, 440 proteins in T4 exhibited significant changes compared to T0, with 299 upregulated and 141 downregulated (Supplementary Figure S1E). When comparing T4 to T2, 297 proteins showed significant differences, with 175 proteins having higher content in T4 and 122 showing lower content in T4 compared to T2 (Supplementary Figure S1F).

**Figure 4:**
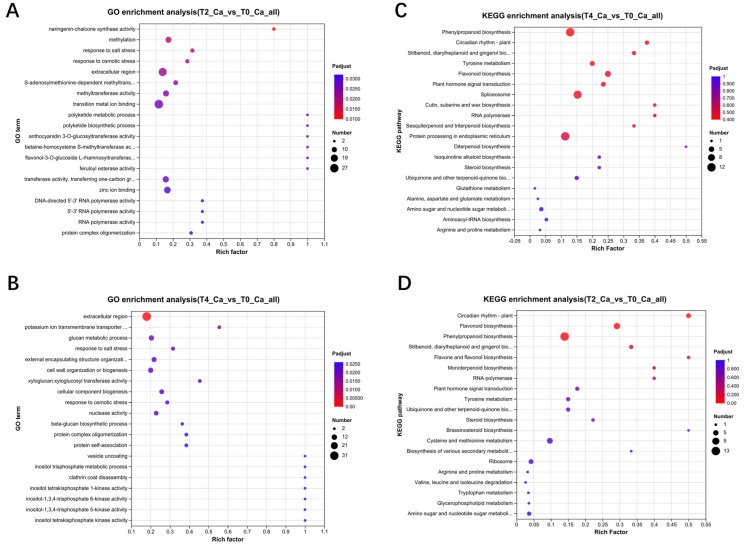
Enrichment analysis of differentially expressed proteins from proteomic data: (**A**,**B**) GO functional enrichment analysis of differentially expressed proteins in T2 and T4 compared to T0; (**C**,**D**) KEGG functional enrichment analysis of differentially expressed proteins in T2 and T4 compared to T0.

Functional enrichment analysis of the proteomic data highlighted key differences between T2 and T0 in GO annotation. Differential proteins in T2 were primarily involved in metabolic processes, mRNA synthesis, and stress responses (Figure 4A). In contrast, proteins in T4 were enriched in membrane vesicle transport, stress response, metabolic processes, and phosphoinositol-related signaling pathways compared to T0 (Figure 4B). KEGG annotation further revealed that the differential proteome of T2 was enriched in functions related to membrane vesicle transport, cytoskeleton organization, and cell wall synthesis (Figure 4C). Conversely, T4’s differential proteome was enriched in cell cycle regulation, reactive oxygen species (ROS)-related metabolism, metabolic enzymes, and transcriptional regulation (Figure 4D).

GSEA analysis of differential proteins between T2 and the control group identified several enriched KEGG pathways, among which were Map00130 (Ubiquinone and other terpenoid quinone biosynthesis) and Map00511 (Other glycan degradation). In contrast, the differential proteome of T4 compared to the control group showed enrichment in Map00130 and Map00670 (One-carbon pool by folate). It is worth noting that Map00511 exhibited significant enrichment between T4 and T2 (Supplementary Figure S3).

### 2.3. Initial Examination of Metabolome Data

In T2 treatment, 811 metabolites exhibited significant variations compared to the control group (T0), with 413 metabolites showing a marked increase and 398 a decrease (Supplementary Figure S1H). Under T4 treatment, 848 metabolites showed notable differences, with 244 metabolites exhibiting a significant increase and 604 a decrease (Supplementary Figure S1I). When comparing T4 and T2, 769 metabolites demonstrated significant changes, with 152 metabolites showing a marked increase and 617 a decrease (Supplementary Figure S1J).

**Figure 5:**
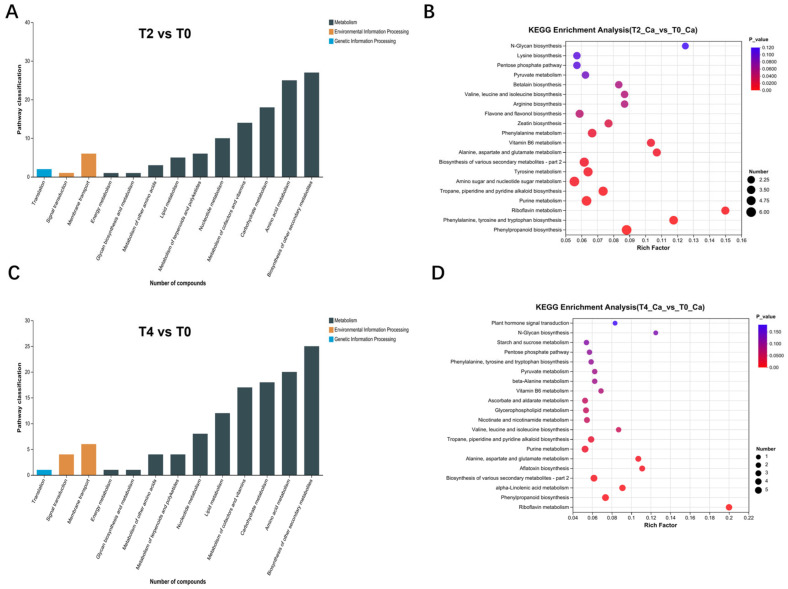
Pathway distribution and KEGG metabolic pathway enrichment analysis of differential metabolites revealed by metabolomic data: (**A**,**B**) pathway distribution and KEGG pathway enrichment analysis of differential metabolites in T2 vs. T0; (**C**,**D**) pathway distribution and KEGG pathway enrichment analysis of differential metabolites in T4 vs. T0.

KEGG annotation of these differential metabolites revealed distinct patterns. Compared to T0, the metabolites were primarily enriched in pathways related to the biosynthesis of secondary metabolites, as well as biological processes such as signal transduction, membrane transport, and translation. The biological processes involving metabolites in T4 (Figure 5C) were similar to those in T2 and T0 (Figure 5A), although with different specific metabolite counts.

**Figure 6:**
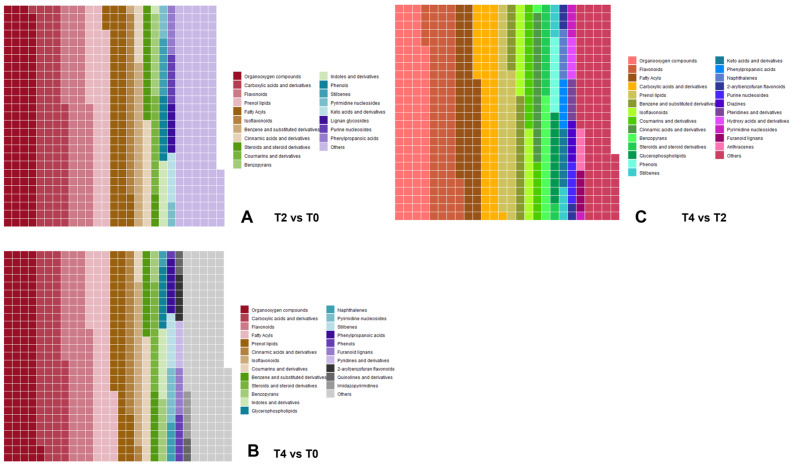
Distribution of differential metabolite categories (classified according to the Human Metabolome Database, HMDB): (**A**) differential metabolites in T2 vs. T0; (**B**) differential metabolites in T4 vs. T0; (**C**) differential metabolites in T4 vs. T2.

The number of differential metabolites between T2 and T0 was generally slightly higher than that between T4 and T0. Furthermore, the differential metabolites between T4 and T0 included an enrichment in plant hormone signaling pathways (Figure 5D), whereas those between T2 and T0 were more prominently enriched in various metabolic processes (Figure 5B). To further explore the differential metabolites across treatments, the Human Metabolome Database (HMDB) was consulted for comparative analysis between T2 and T0 (Figure 6A), T4 and T0 (Figure 6B), as well as T4 and T2 (Figure 6C). The distribution of various metabolite types is shown in Figure 6.

### 2.4. Multi-Omics Data Analyses

**Figure 7:**
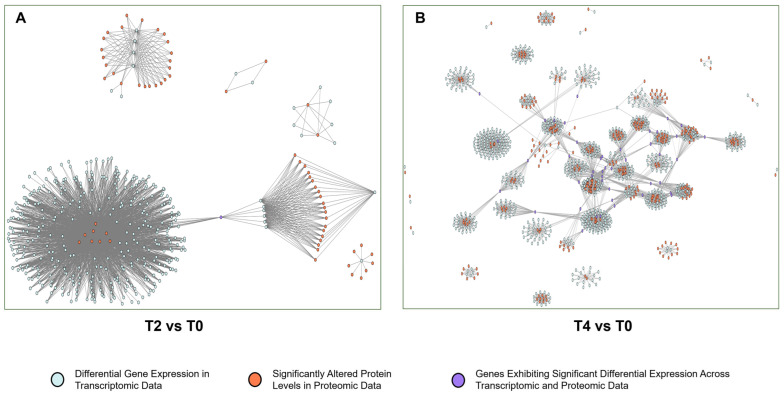
PPI networks of differentially expressed genes: (**A**) gene co-expression correlation network for T2 vs. T0; (**B**) gene co-expression correlation network for T4 vs. T0.

**Figure 8:**
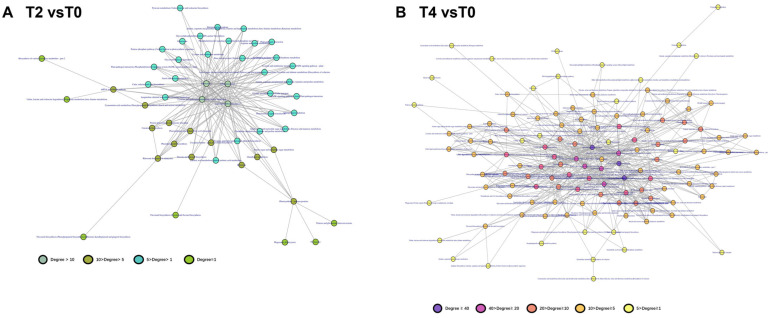
Correlation network of gene functional modules constructed based on expression correlation networks: (**A**) functional module correlation network for differentially expressed genes in T2 vs. T0; (**B**) functional module correlation network for differentially expressed genes in T4 vs. T0.

Transcriptome and proteome data were integrated to construct PPI networks for differentially expressed genes and proteins (T2 vs. T0, Figure 7A) under the various treatments (T4 vs. T0, Figure 7B). The network analysis revealed that genes with significant differences in both protein content and gene expression acted as central nodes within multiple modules, forming a complex interaction map. To streamline interpretation, these genes were categorized into functional modules, leading to the creation of a functional module network based on the original structure (T2 vs. T0, Figure 8A; T4 vs. T0, Figure 8B).

**Figure 9:**
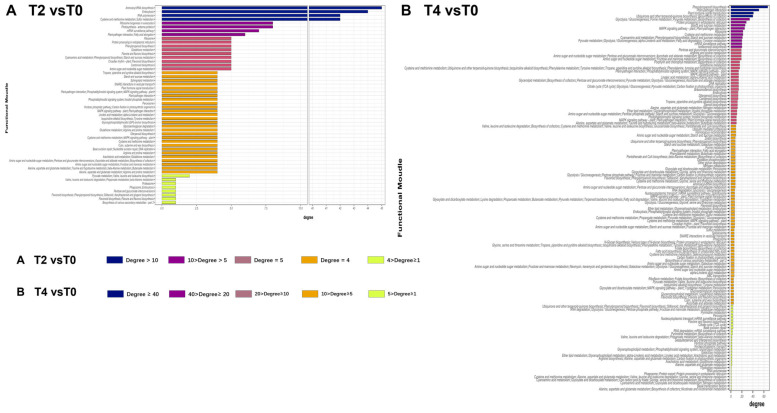
Distribution of network connectivity degrees for functions within gene functional module correlation networks: (**A**) connectivity degree distribution of functions in the functional module network for T2 vs. T0; (**B**) connectivity degree distribution of functions in the functional module network for T4 vs. T0.

The degree of each function within this network was quantified to assess its relative importance under calcium treatment. The analysis revealed that under T2 treatment, four functional modules with high connectivity were identified: aminoacyl-tRNA biosynthesis, endocytosis, RNA polymerase activity, and sulfur metabolism (cysteine and methionine metabolism) (Figure 9A). In contrast, under the higher calcium concentration of T4, additional specialized metabolic and functional modules were affected, including plant-pathogen interactions, plant hormone signal transduction, and ubiquinone/terpenoid-quinone biosynthesis (Figure 9B).

## 3. Discussion

Our experiments demonstrated that calcium ion treatment at varying concentrations exerts a dual effect on the growth and development of tissue-cultured seedling roots. Specifically, an optimal concentration promotes root growth and development, while higher concentrations inhibit it. Early studies on plant development have revealed an antagonistic interaction between calcium and auxin, with calcium inhibiting stem growth while promoting root development [21]. The growth phenotype observed in tissue-cultured seedlings under T2 calcium treatment supports this role of calcium. In contrast, higher calcium concentrations in the T4 treatment inhibited root growth, likely due to calcium’s regulatory role in the cell wall [4]. All calcium ion treatments led to an increase in matrine content, whereas oxymatrine content only increased at specific concentrations and decreased at higher concentrations. Moreover, trifolirhizin and Maackiain contents significantly increased compared to the control, but only under high calcium ion concentrations (T3, T4).

The opposite trends observed between the T2 (promotive) and T4 (inhibitory/shift-inducing) treatments in both processing phenotype and omics responses are highly consistent with the well-documented Ca^2+^ signaling biphasic effect, also known as hormesis, in plants [13]. At optimal physiological concentrations (T2), the mild and transient increase in cytosolic Ca^2+^ concentration ([Ca^2+^]*_cyt_*) acts primarily as an essential nutrient signal, promoting growth, cell expansion, and maintaining normal metabolic flux. Conversely, at supra-optimal or stress-inducing concentrations (T4), the Ca^2+^ influx becomes larger and more sustained, transitioning the signal into a non-biotic stress cue [22]. This shift triggers a fundamental resource allocation trade-off: resources are redirected from growth support (leading to root inhibition) towards defense-related secondary metabolism [23]. Specifically, the significant accumulation of defense-related compounds like trifolirhizin and maackiain under T4 treatment represents the cell’s strategy to prioritize defense pathways over biomass accumulation in response to the perceived high-calcium stress. This demonstrates that the concentration of Ca^2+^ acts as a precise switch, determining whether the plant adopts a growth-prioritizing (T2) or a defense-prioritizing (T4) metabolic state.

The regulatory mechanisms by which calcium influences specific components in medicinal plants remain unclear, with occasional studies suggesting calcium’s definitive effects on certain compounds [24,25,26]. It is well-established, however, that calcium affects various functions, including stress responses [27] and plant hormone signaling pathways [28], which are linked to secondary metabolism and may influence the synthesis of active compounds in medicinal plants. This indicates that calcium ion treatment exerts a multifaceted effect on the accumulation of medicinal substances, simultaneously suppressing the synthesis of some active components while enhancing that of others.

The PPI network derived from altered genes and proteins is highly complex (Figure 7), complicating the identification of key genes and pathways through which calcium ions exert their effects. Discerning a single significant gene or protein pathway from the multi-omics data remains challenging. To address this, genes and proteins were categorized based on their functional roles, grouping those with similar functions into modules within the transcriptional regulatory network. A network of these functional modules was then generated to simplify analysis. The modular network (Figure 8) reveals a complex interaction of functional modules. To clarify the role of each module, functional pathways were ranked by node degree within the network (Figure 9). Analysis of the module network identified that, under T2 calcium treatment, aminoacyl-tRNA biosynthesis and RNA polymerase were likely involved in regulating gene transcription and translation, while endocytosis appeared to be associated with intracellular vesicle transport. These findings suggest that calcium signaling primarily influences gene expression and cellular state.

Unexpectedly, sulfur metabolism also emerged as a key player in calcium treatment. This finding suggests a critical intersection between Ca^2+^ signaling and thiometabolism, warranting further mechanistic speculation. Relevant literature indicates a tight physiological link between calcium and sulfur [29]. It is proposed that calcium may influence the availability of sulfate or regulate key enzymes in the sulfur assimilation pathway (e.g., ATP sulfurylase, O-acetylserine (thiol) lyase).

A key finding is that the products of sulfur metabolism are central to the cell’s response to the high-calcium stress observed in the T4 treatment. Sulfur is a vital component of glutathione (GSH), the primary non-enzymatic antioxidant. Enhanced sulfur metabolism would be required to replenish the GSH pool, thereby maintaining redox homeostasis and detoxifying reactive oxygen species (ROS) generated during the perceived high-calcium stress [30,31]. This redox control is essential for plant survival under stress. This strongly indicates that the upregulation of sulfur metabolism under high Ca^2+^ is part of a coordinated defense-oriented metabolic shift. Given sulfur’s critical role in plant growth, stress response, hormone signaling, and secondary metabolism, the regulation of sulfur metabolism by calcium warrants further investigation.

As the calcium concentration increased under T4 treatment, additional specific metabolic functions were affected. Thus, calcium likely impacts physiological functions such as gene transcription, vesicle transport, and sulfur metabolism at moderate concentrations, with broader metabolic processes influenced at higher concentrations.

**Figure 10:**
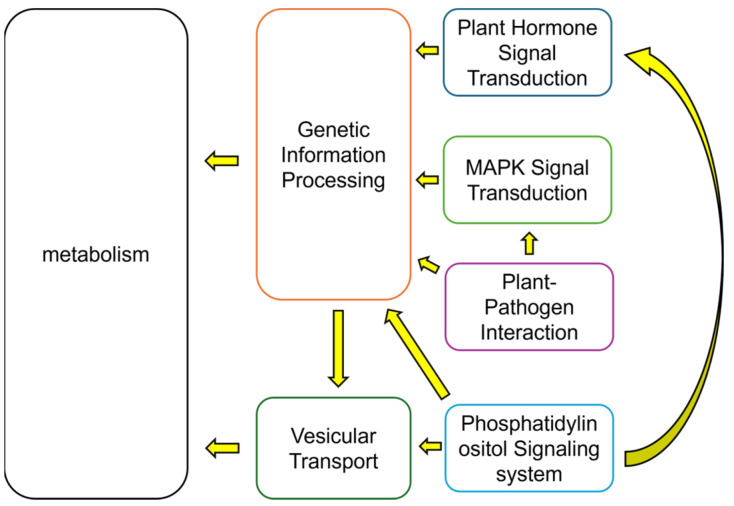
Proposed mechanism of calcium-mediated regulation of metabolic function: Calcium influences plant metabolic activity and regulates the synthesis of bioactive compounds through pathways including plant hormone signal transduction, MAPK signaling, phosphatidylinositol signaling system, plant–pathogen interaction, vesicular transport, and genetic information processing.

Both T4 and T2 treatments have revealed several key signaling pathways that play significant roles under calcium treatment. These include pathways involved in plant-pathogen interactions, the MAPK signaling pathway, plant hormone signal transduction, and the phosphatidylinositol signaling transduction pathway. In addition to these four signaling pathways, most other functional modules are associated with metabolic processes, with a smaller proportion related to genetic information processing and vesicular transport. The interrelationships among these major functions are shown in Figure 10. Specifically, signal transduction affects gene expression and translation (genetic information processing), which in turn influences vesicular transport and metabolic status. Moreover, vesicular transport may also impact metabolic pathways. Calcium’s role in these functional modules appears to be primarily involved in the signal transduction phase, where calcium ions act as critical second messengers. Calcium is essential in the signal transduction triggered by plant–pathogen interactions, with both calcium and the MAPK signaling pathway being implicated in plant immunity [32]. A study by Boudsocq et al. [33] demonstrated that the Ca^2+^-related CDPK signaling pathway shares common targets with downstream components of the MAPK pathway in immunity-related signaling.

Further data analysis suggests that calcium treatment affects the plant phospholipid signaling pathway, which in turn regulates vesicular transport and influences plant hormone actions within cells. Given the close relationship between this pathway and plant growth and development, calcium likely plays a role in modulating this signaling system, thereby affecting signal transmission [34].

This study constructed a modular network of calcium-regulated growth and secondary metabolism in medicinal plants through integrated multi-omics analysis and identified several key functional modules and candidate genes. Although in planta functional validation of individual genes was not performed here, the network and core pathways identified (e.g., plant hormone signaling, MAPK, phospholipid signaling, and sulfur metabolism) provide clear targets for future functional studies. Subsequent research could employ genetic transformation, gene editing, or other functional genomics approaches to validate the roles of key candidate genes (e.g., those involved in calcium sensing, sulfur assimilation, or defense compound biosynthesis) identified in this work, thereby further elucidating their precise molecular mechanisms.

In conclusion, the broad functional categorization of these signaling pathways, along with their interactions with metabolism and vesicular transport, is depicted in Figure 10. Based on these findings, particular focus should be directed toward the signaling pathways and sulfur metabolism when investigating calcium’s effects on the growth and development of *S. tonkinensis* and the synthesis of its bioactive compounds. Given the complexity of the interactions between these pathways and metabolic processes, further investigation is needed. A metabolic systems analysis utilizing the existing metabolome data will be instrumental in clarifying the relationships between these metabolic processes and the synthesis of bioactive substances.

## 4. Materials and Methods

### 4.1. Materials

The tissue culture seedlings used in this study were sourced from the specialized ex situ collection at the Guangxi Botanical Garden of Medicinal Plants. These materials, originally derived from a wild population in Guangxi, were selected from several bottles containing well-developed *S. tonkinensis* tissue culture seedlings. The seedlings were cut into 2–3 cm stem segments and inoculated onto a new propagation medium (MS + 5.0 mg·L^–1^ 6-BA + 0.3 mg·L^–1^ IAA + 0.3 mg·L^–1^ KT) to induce bud proliferation. Upon the formation of clustered buds, the seedlings were transferred to a fresh medium (MS + 1.0 mg·L^–1^ IAA) for further cultivation. Seedlings with enhanced lignification were selected, and apical stem segments with leaves were trimmed to 2–3 cm. These segments were then planted into 1/2 MS medium with varying calcium concentrations for rooting culture, using sterile forceps. Each bottle contained 8 seedlings, and 30 bottles were prepared for each treatment group. Six different calcium concentrations were applied (T0, T0.5, T1, T2, T3, T4, as shown in Table 1). The rooting medium consisted of 30 g·L^–1^ sucrose, 3.4 g·L^–1^ agar, with a pH of 5.8. Cultures were maintained at 25 °C under a light intensity of 1500–2000 lux and a 12–14 h photoperiod. After 60 days of calcium stress treatment, samples were collected for the analysis of growth, root traits, aboveground development, and medicinal compound content.

### 4.2. Multi-Omics and Statistical Analyses

Omics data analysis was primarily conducted on the Majorbio cloud platform (https://cloud.majorbio.com) [35]. Data processing was performed in R (version 4.3.2), using the Tidyverse package [36] for data organization, the igraph package (version 2.1.4) [37] for network analysis and visualization, the Pathview package [38] for KEGG pathway visualization, and the GseaVis package(version 0.1.1) [39] for GSEA result visualization. GSEA analysis was carried out using GSEA 4.3.2 software [40,41].

### 4.3. Determination of Growth Indicators

Growth indicators, including plant height, stem diameter, leaf count, rooting rate, root length, and root dry weight, were assessed through statistical analyses using one-way ANOVA and the LSD method (α < 0.05).

Plant height was measured as the maximum height of the main stem with a ruler. Stem diameter, defined as the thickest part of the lower stem, was measured using a vernier caliper. Leaf count was determined by counting the proliferating leaves. Rooting rate was calculated as the ratio of rooted explants to total inoculated explants. Root length was measured with a ruler, and root dry weight was determined by drying roots to a constant weight in an oven before measurement.

### 4.4. Determination of Pharmacological Substances

#### 4.4.1. Determination of Matrine and Oxymatrine

The contents of matrine and oxymatrine were quantified according to the methods specified in the 2020 edition of the Pharmacopoeia of the People’s Republic of China.

Extraction Method: To 0.1 g of the sample, 2 mL of extraction solution (methylene chloride: methanol: ammonia, 40:10:1) was added. The mixture was left at room temperature for 30 min, followed by sonication for 30 min and centrifugation at 4000 rpm for 10 min at room temperature. The supernatant was then collected, and 1 mL was evaporated to dryness under nitrogen gas at 40 °C. The dried residue was reconstituted in 1 mL of methanol and filtered through a 0.22 μm syringe filter for subsequent analysis.

HPLC Conditions: HPLC analysis was conducted using a Shimadzu LC-2030 Plus system (Shimadzu, Kyoto, Japan) equipped with an Agilent Polaris 5 NH2 column (5 μm, 250 × 4.6 mm). The mobile phase consisted of acetonitrile, isopropanol, and 3% phosphoric acid solution in a ratio of 80:5:15. The injection volume was 5 μL, the flow rate was set at 0.5 mL/min, and the column temperature was maintained at 25 °C. Detection was performed at 210 nm.

#### 4.4.2. Determination of Maackiain and Trifolirhizin

Extraction Method: A 0.1 g sample was weighed, mixed with 1 mL of methanol, and ground thoroughly. The mixture was subjected to ultrasonication for 50 min, and then the volume was adjusted back to the original level with methanol. After centrifuging the sample at 4000 rpm for 10 min, the supernatant was collected and filtered through a 0.22 μm membrane for further analysis.

HPLC Conditions: The HPLC analysis was performed using the same Shimadzu LC-2030 Plus system with an Agilent Plus C18 column (5 μm, 250 × 4.6 mm). The mobile phase consisted of acetonitrile and water, following a gradient program: starting with 25% acetonitrile for 5 min, increasing to 50% over 50 min, then rising to 95% over 25 min. The system was held at 95% acetonitrile for 6 min and then rapidly returned to 25% within 1 min and equilibrated for 5 min. Detection was conducted at 205 nm with a 10 μL injection volume, a flow rate of 1 mL/min, and a column temperature of 30 °C.

### 4.5. Collection of Omics Data

Omics data, including transcriptome, proteome, and metabolome, were collected from tissue-cultured seedlings subjected to T0, T2, and T4 treatments. The number of biological replicates was determined based on the technical characteristics and common practices for each omics platform. Both transcriptomic and proteomic analyses (n = 3 biological replicates per group) typically exhibit high technical reproducibility, and three replicates are widely considered sufficient for robust statistical power in discovery-phase studies. In contrast, metabolomic profiles are more dynamic and susceptible to subtle biological variations; therefore, a larger number of replicates (n = 6 per group) was employed to ensure greater statistical reliability and to accurately capture the metabolic variability.

#### 4.5.1. Transcriptome Data Collection

Samples from the T0, T2, and T4 treatment groups, each comprising three biological replicates, were utilized for transcriptome sequencing. RNA extraction and sequencing were performed at Shanghai Majorbio, China, with genomic data for *S. tonkinensis* provided by the Guangxi Botanical Garden of Medicinal Plants to support the analysis.

mRNA sequencing was conducted on the Illumina Novaseq 6000 platform, following the Illumina Truseq™ RNA sample preparation kit protocol for library construction.

Raw paired-end reads were processed with fastp [42] to trim low-quality sequences, using default parameters. Clean reads were aligned to the reference genome in orientation mode with HISAT2 [43]. Mapped reads were then assembled using StringTie [44] in a reference-based approach. Transcript expression levels were quantified using the transcripts per million reads (TPM) method, based on read mapping to the reference genome via RSEM [45]. Differential expression analysis was performed with DESeq2 [46], and differentially expressed genes (DEGs) were identified using a threshold of |log_2_FC| ≥ 1 and an adjusted *p*-value (FDR) < 0.05. GO functional enrichment analysis was executed using Goatools, and KEGG pathway analysis was conducted via KOBAS [47].

#### 4.5.2. Protome Data Collection

Protein samples from the T0, T2, and T4 groups, each with three biological replicates, were submitted to Shanghai Majorbio (China) for protein extraction and proteomic analysis. Protein extraction was followed by enzymatic digestion using trypsin, and data were collected using an EASY nLC-1200 system (Thermo Fisher Scientific, Waltham, MA, USA) coupled with a timsTOF Pro2 mass spectrometer (Bruker, Billerica, MA, USA). MS/MS spectra were analyzed with MaxQuant version 2.0.3.1 against a self-built database constructed from genomic data provided by MajorBio. Peptides were identified based on the highest-scoring match to predicted masses in the database. Bioinformatic analysis of proteomic data was performed on the Majorbio Cloud platform (https://cloud.majorbio.com). Differential protein analysis was performed using a *t*-test, and differentially expressed proteins (DEPs) were defined by a combination of |log_2_FC| ≥ 1 and an unadjusted *p*-value < 0.05. GO (http://geneontology.org/ accessed on 24 October 2024) and KEGG (http://www.genome.jp/kegg/ accessed on 25 October 2024) were used for functional annotation, and protein–protein interaction (PPI) analysis was conducted using String v11.5.

The functional module networks were derived from the original protein-protein interaction (PPI) networks of differentially expressed genes and proteins. The construction process was as follows: First, each node (gene/protein) in the PPI network was assigned to a specific functional module based on its Kyoto Encyclopedia of Genes and Genomes (KEGG) pathway annotations. Subsequently, a new network was generated where these functional modules themselves became the nodes. An edge was established between two functional modules if there was at least one interaction between any constituent node from one module and any node from the other in the original PPI network. This process effectively aggregated the complex PPI network into a simplified functional module network, revealing higher-level interactions between biological processes. Finally, redundant edges were removed, and the network was visualized using the igraph package in R.

#### 4.5.3. Metabolomics Data Collection

Tissue-cultured seedlings treated with T0, T2, and T4 were frozen in liquid nitrogen and sent to Shanghai Majorbio (Shanhai, China) for metabolomics analysis, with six biological replicates per treatment. To initiate the extraction process, 50 mg of the sample was placed in a 2 mL centrifuge tube, and 400 μL of extraction solution (methanol–water = 4:1 *v*/*v*) containing 0.02 mg/mL of the internal standard (L-2-chlorophenylalanine) was added. After grinding, metabolites were extracted, and equal volumes from all samples were combined to prepare quality control (QC) samples. A QC sample was inserted into the analysis every 5–15 samples to assess the reproducibility of the entire process. LC-MS/MS and non-targeted metabolomics data collection were performed using Thermo Fisher’s ultra-high-performance liquid chromatography–tandem Fourier transform mass spectrometry (UHPL-Q Exact HF-X) system. Raw LC-MS data were processed using Progenesis QI (Waters Corporation, Milford, MA, USA) for baseline filtering, peak identification, integration, retention time correction, and peak alignment, resulting in a data matrix containing retention time, mass-to-charge ratio, and peak intensity. The matrix was pre-processed by retaining metabolic features detected in at least 80% of samples. For specific metabolites below the lower limit of quantification, the minimum value was imputed, and each metabolic signature was normalized to the sum. To account for errors due to sample preparation and instrument instability, peak intensities were normalized using the sum normalization method, generating the normalized data matrix. Variables with a relative standard deviation (RSD) > 30% in QC samples were excluded, and the data were logarithmically transformed (log10) to obtain the final matrix for subsequent analysis. Significantly different metabolites were identified based on VIP values > 1 and *p*-values < 0.05, derived from the OPLS-DA model and Student’s *t*-test. Additionally, MS and MS/MS mass spectrometry data were compared against the HMDB (http://www.hmdb.ca/ accessed on 17 May 2024) and Metlin (https://metlin.scripps.edu/ accessed on 20 May 2024) public databases, as well as a proprietary self-built database, to identify metabolites.

### 4.6. Real-Time PCR

To validate the transcriptome sequencing results, real-time quantitative PCR (RT-qPCR) was conducted on twelve genes. RNA was extracted from the T0, T2, and T4 samples using the FastPure Plant Total RNA Isolation Kit (Polysaccharides & Polyphenolics-rich) (RC411-01, Vazyme, Nanjing, China). The isolated RNA was subsequently reverse-transcribed into complementary DNA (cDNA) using the HiScript III RT SuperMix for qPCR (+gDNA wiper) (R323-01, Vazyme, Nanjing, China). Primer sequences for the qRT-PCR analysis are provided in the Supplementary Materials (Table S1). The LY-3745 gene was used as the internal reference, and qPCR was performed using the ChamQ Universal SYBR qPCR Master Mix (Q711-02, Vazyme, Nanjing, China).

## Figures and Tables

**Table 1:** Each medium contains varying concentrations of calcium, along with 30 g·L^−1^ sucrose and 3.4 g·L^−1^ agar, adjusted to a pH value of 5.8.

Treatments	Calcium concentration
T0	Ca^2+^ 0 mmol·L^−1^
T0.5	Ca^2+^ 0.748 mmol·L^−1^
T1	Ca^2+^ 1.495 mmol·L^−1^
T2	Ca^2+^ 2.99 mmol·L^−1^
T3	Ca^2+^ 4.485 mmol·L^−1^
T4	Ca^2+^ 5.98 mmol·L^−1^

**Table 2:** Explanation of KEGG pathway identification in gene set enrichment analysis (GSEA).

KEGG Pathway ID	KEGG Pathway
Map00196	Photosynthesis—antenna proteins
Map04626	Plant-pathogen interaction
Map00040	Petose and glucuronate interconversions
Map00941	Flavonoid biosynthesis
Map00531	Glycosaminoglycan degradation
Map03010	Ribosome
Map03050	Proteasome
Map00280	Valine, leucine and isoleucine degradation
Map04016	MAPK signaling pathway—plant
Map00940	Phenylpropanoid biosynthesis
Map00480	Glutathione metabolism
Map00710	Carbon fixation in photosynthetic organisms
Map00061	Fatty acid biosynthesis
Map00030	Pentose phosphate pathway
Map00944	Flavone and flavonol biosynthesis

## Data Availability

All relevant analytical data generated or analyzed during this study are included in this published article and its Supplementary Materials. The raw omics datasets have been deposited in the ScienceDB database under restricted access (doi:10.57760/sciencedb.30082). Researchers who wish to access these raw datasets must comply with the data access conditions specified in the ScienceDB repository. Access can be granted upon reasonable request and with approval from the corresponding author.

## Supplementary Materials

The following supporting information can be downloaded at: https://www.mdpi.com/article/10.3390/plants15010133/s1, Figure S1: Volcano plots displaying data distribution across transcriptomic, proteomic, and metabolomic analyses. (A–C) Volcano plots of transcriptomic data; (D–F) Volcano plots of proteomic data; (G–J) Volcano plots of metabolomic data; Figure S2: GSEA results of differentially expressed genes from transcriptomic data. (A) GSEA of differentially expressed genes in T2 vs. T0; (B) GSEA of differentially expressed genes in T4 vs. T0; (C) GSEA of differentially expressed genes in T4 vs. T2. Figure S3: GSEA results of differentially expressed proteins from proteomic data. (A) GSEA of differentially expressed proteins in T2 vs. T0; (B) GSEA of differentially expressed proteins in T4 vs. T0; (C) GSEA of differentially expressed proteins in T4 vs. T2. Table S1: List of genes and primers used in RT-PCR.

## Abbreviations

The following abbreviations are used in this manuscript:

DEGs	Differentially Expressed Genes
GO	Gene Ontology
KEGG	Kyoto Encyclopedia of Genes and Genomes
GSEA	Gene Set Enrichment Analysis
ROS	Reactive Oxygen Species
HMDB	Human Metabolome Database
HPLC	High Performance Liquid Chromatography
IAA	3-Indoleacetic acid
KT	Kinetin 6-Furfurylaminopurine
MAPK	Mitogen-activated Protein Kinase
TPM	Transcripts Per Million
