# Supplementary figures and images for: Integrated Omic Analyses Reveal Module Networks Regulating Growth and Bioactive Component Synthesis of Sophora tonkinensis via Calcium Modulation
Source: Plants (Basel). 2026 Jan 2;15(1):133. doi: 10.3390/plants15010133 (PMC12787832; doi:10.3390/plants15010133)

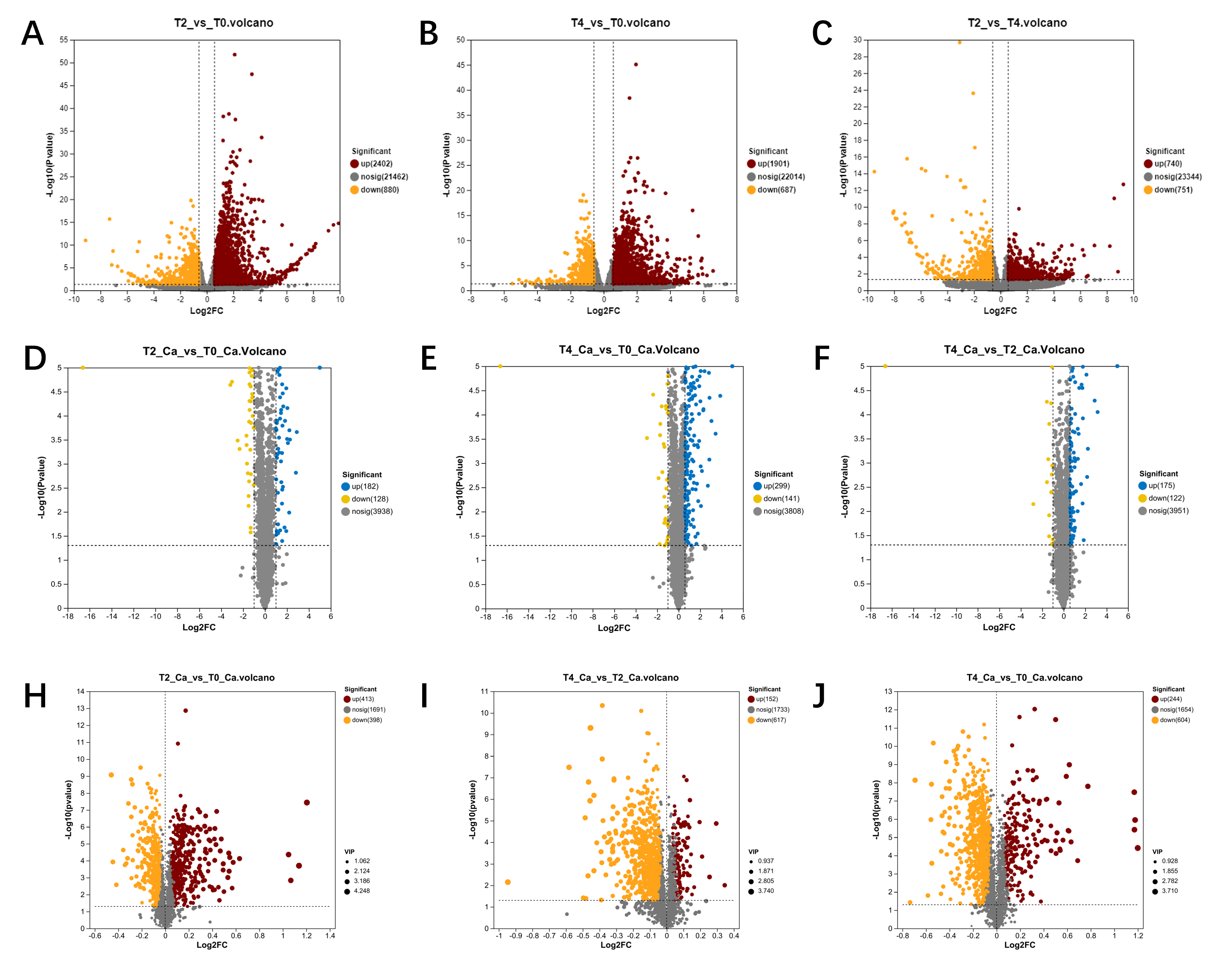

Supplement: Supplementary file 1 [file plants-15-00133-s001.zip › Figure S1.tif]

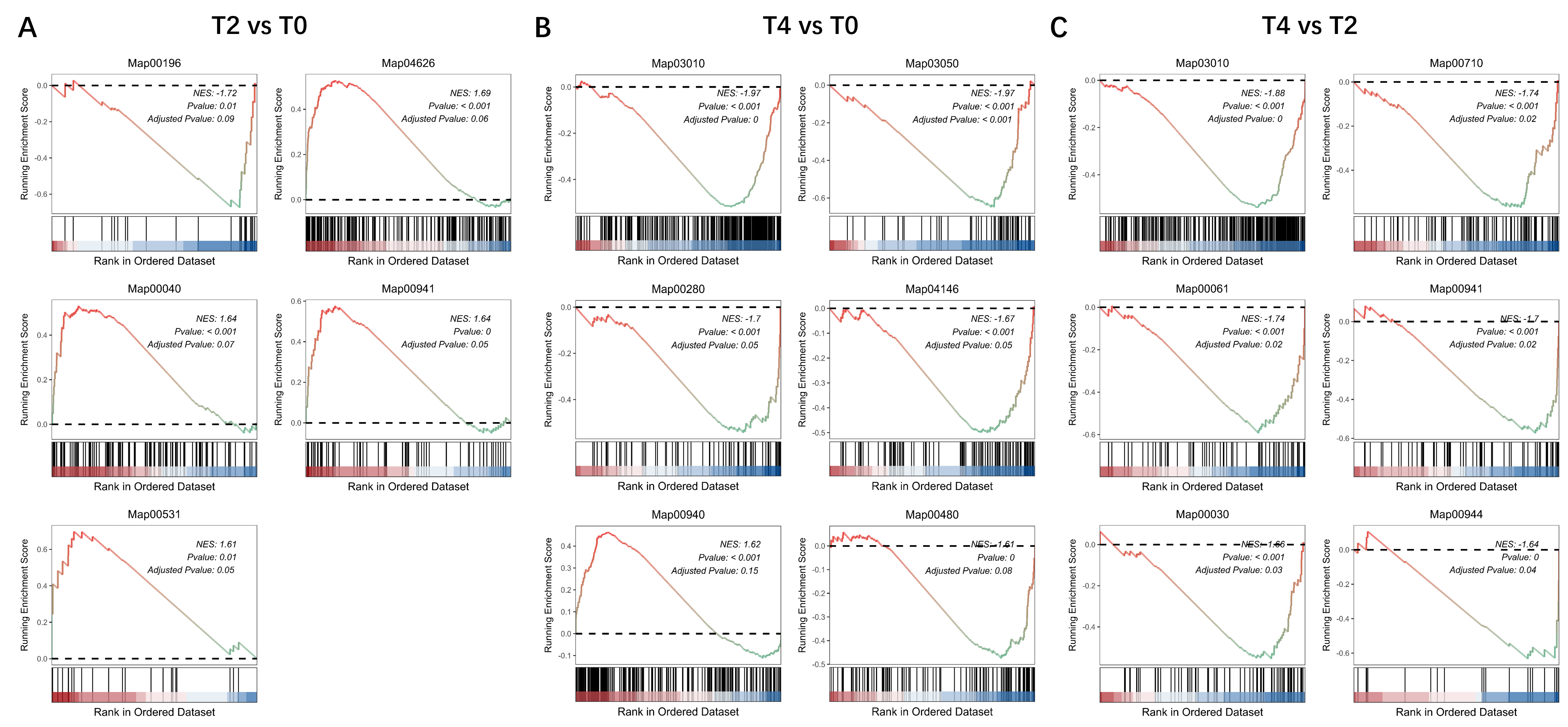

Supplement: Supplementary file 1 [file plants-15-00133-s001.zip › Figure S2.tif]

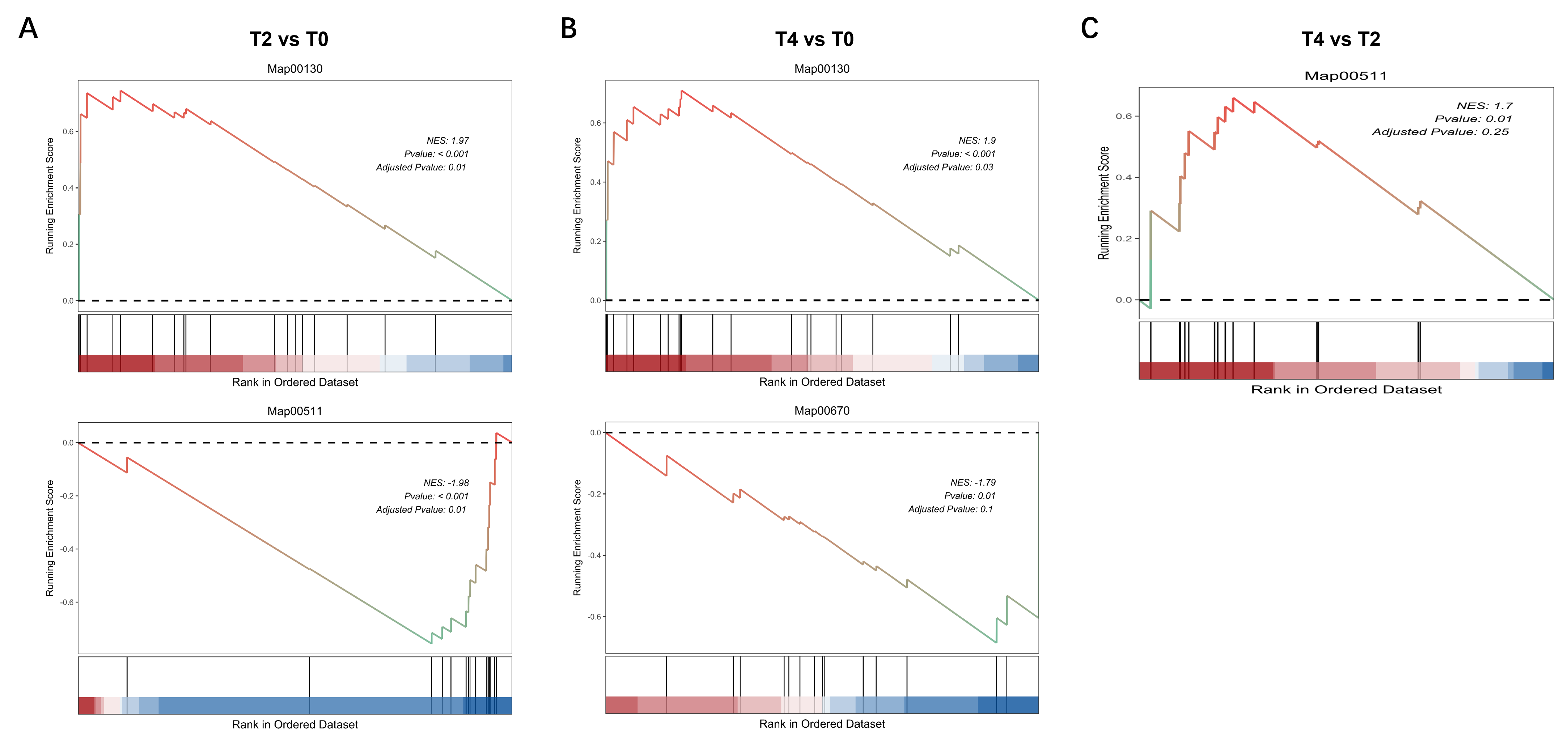

Supplement: Supplementary file 1 [file plants-15-00133-s001.zip › Figure S3.tif]
